# Eco-Friendly Semi-Interpenetrating Polymer Network Hydrogels of Sodium Carboxymethyl Cellulose/Gelatin for Methylene Blue Removal

**DOI:** 10.3390/ma16093385

**Published:** 2023-04-26

**Authors:** Rongbin Chen, Shanbin Yang, Bing Liu, Youlin Liao

**Affiliations:** Engineering Research Center of Biotechnology of Active Materials (Ministry of Education), College of Chemistry, Chongqing Normal University, Chongqing 401331, China

**Keywords:** semi-interpenetrating, hydrogel, sodium carboxymethyl cellulose, gelatin, citric acid

## Abstract

The present work describes the potential application of environmentally friendly sodium carboxymethylcellulose/gelatin (CMC/Gel) semi-interpenetrating hydrogels prepared by citric acid as a nontoxic cross-linking agent to adsorb dyes. The prepared hydrogels were characterized by X-ray diffraction (XRD), Fourier transform infrared spectroscopy (FT-IR), scanning electron microscopy (SEM), thermogravimetric analysis (TGA/DTG), and swelling study. The adsorption performance of CMC/Gel2 (C/G2) hydrogel on methylene blue (MB) was investigated. The results showed the better adsorption conditions: adsorption time of 300 min, initial MB concentration of 500 mg/L, adsorbent dosage of 1.2 g/L, solution pH of 7, and temperature of 30 °C. The adsorption kinetics fit the pseudo-second order kinetics model, and the adsorption isotherm fit the Langmuir isotherm model. The maximum adsorption capacity reached 943.40 mg/g. The adsorption process is a spontaneous exothermic process. After three adsorption–desorption cycles, the removal rate of MB by hydrogel still reached 85%, with good reusability. Consequently, the hydrogel can be used as an environmentally friendly, stable, and efficient adsorbent for dyes in wastewater treatment.

## 1. Introduction

As social economy advances progressively, environmental issues are becoming increasingly paramount to people, and the harm spawned from wastewater pollution to the ecological environment and human health is capturing increasingly widespread concern [1]. Methylene blue is a cationic dye with a stable structure [2], and it has been extensively used in textile dyeing and printing industry. In dye wastewater, it is a major source of pollution. To effectively deal with the pollution induced by dye wastewater, new technologies and methods of wastewater treatment such as electrolytic coagulation, chemical oxidation, electrochemical method, photocatalytic method, air floatation, membrane separation, and biodegradation are constantly put forth [3,4,5,6,7,8,9]. Nonetheless, despite the fact that these treatment methods are more effective, they have strict process conditions, unsatisfactory stability, and high treatment cost. As a wastewater treatment method with easy operation, high treatment efficiency and speed, the adsorption method is more economical and efficient, provided that new pollutants are not introduced. Furthermore, this method has gained the attention of researchers. In fact, some common adsorbents are costly to produce and may trigger secondary pollution [10]. Hence, the development of new dye adsorbents with high efficiency, low cost, and environmental friendliness is receiving increasingly extensive attention from researchers.

In general, hydrogel is a polymer with hydrophilic groups and a three-dimensional network structure, which can swell speedily in aqueous solution without being dissolved. Furthermore, as a considerably promising functional material, it is universally employed in agricultural water retention, biomedicine, sewage treatment, and other fields [11]. The special functional groups of the hydrogel network can interact with dye molecules. In such a case, it can be employed as a new adsorbent to quickly adsorb and separate organic dyes, effectively coping with the problem of dye wastewater treatment [12]. In particular, hydrogels prepared from natural polymers, which are abundant and renewable in nature, are non-toxic and biocompatible, and can be biodegraded [13]. As an efficient and low environmental impact adsorbent, it can lessen environmental pollution while improving resource utilization, and has been universally investigated by researchers.

Sodium carboxymethyl cellulose (CMC) is a natural hydrogel raw material and is known as “industrial MSG” [14]. It has degradability, hygroscopicity, and satisfactory biocompatibility. Meanwhile, the large amount of -COOH and -OH on the surface of CMC provides many active sites for the adsorption of pollutants. In comparison, gelatin is a water-soluble polymeric peptide polymer with desirable biocompatibility and degradability [15]. It is a typical amphiphilic polymer electrolyte, for the reason that -COOH and -NH_2_ on its molecular chain will dissociate/hydrolyze into -COO^−^ and -NH_3_^+^ when dissolved in water, which can react with dyes. Under such circumstances, gelatin composite hydrogels can be used to remove dye from wastewater. Citric acid is a non-toxic cross-linking agent that can be used to prepare cellulose derivative hydrogels [16]. At high temperature, citric acid forms an anhydride and reacts with the hydroxyl groups of adjacent components to form ester cross-links between the polymer chains of the cellulose derivative [17].

In a previous study, Nádia et al. used citric acid cross-linked carboxymethyl cellulose to prepare hydrogel membranes for MB adsorption [18], which not only exhibited undesirable structural strength but also unsatisfactory adsorption efficiency, as the maximum adsorption capacity was 25 mg/g. Ikrame’s research team used citric acid cross-linked sodium carboxymethyl cellulose and hydroxyethyl cellulose to prepare hydrogel membranes for MB removal [19], and the maximum adsorption capacity was 769.23 mg/g. In this study, we prepared previously unreported environmentally friendly sodium carboxymethylcellulose/gelatin semi-interpenetrating hydrogels by the solution casting method. The prepared hydrogels were characterized using X-ray diffraction (XRD), thermogravimetric analysis (TGA/DTG), Fourier transform infrared spectroscopy (FT-IR), and scanning electron microscopy (SEM). Furthermore, the swelling behavior of the prepared hydrogels was investigated at various salt ion concentrations and diverse pH. The best hydrogel was selected by the above characterization for conducting the adsorption performance study on MB. Subsequently, the adsorption performance of the hydrogel on MB was investigated at a diverse time, adsorbent dosage, pH, temperature, and initial MB concentration to evaluate in depth the adsorption capacity of the cross-linked sodium carboxymethylcellulose/gelatin semi-interpenetrating hydrogels for the removal of MB from wastewater.

## 2. Materials and Methods

### 2.1. Materials

Gelatin (Gel) was purchased from Chendu Huaxia Chemical Reagent Co., Ltd. (Chengdu, China). Sodium carboxymethylcellulose (CMC, viscosity 5000–15,000 mPa.s) was purchased from Shanghai Macklin Biochemical Co., Ltd. (Shanghai, China). Citric acid (CA, anhydrous), hydrochloric acid (HCl), sodium chloride (NaCl), and sodium hydroxide (NaOH) were purchased from Chengdu Kelon Chemical Reagent Co., Ltd. (Chengdu, China). Methylene blue (MB) was purchased from Tianjin Zhiyuan Chemical Reagent Co., Ltd. (Tianjin, China). Deionized water was used to prepare solutions, and all chemical reagents were of analytic grade.

### 2.2. Hydrogels Preparation

On the basis of the method described by Marta et al. [20], semi-interpenetrating polymer network hydrogels of sodium carboxymethyl cellulose/gelatin were first prepared and the appropriate changes were made. To be specific, 3 g CMC and 1 g gelatin were respectively dissolved in 100 mL deionized water and stirred at 40 °C for 2 h. Subsequently, the gelatin solution was added to the CMC solution. Afterwards, 0.135 g CA was added and stirred at 300 rpm for 1.5 h. After that, the solution was left overnight without stirring and the air bubbles were removed. The homogeneous solution was poured into the petri dish with a diameter of 9 cm and dried at 50 °C in the oven for 48 h. The dried CMC/Gel xerogel was cured in the oven at 120 °C for 8 h, and the cross-linking reaction was promoted between the carboxymethyl cellulose molecular chains. The cross-linked products after the reaction were soaked in an appropriate amount of deionized water for 24 h to remove the remaining cross-linker. Finally, the hydrogel was dried in an oven at 40 °C for 48 h, and we named the modified hydrogel sample C/G1. The above steps were repeated to prepare other hydrogels to be in line with the experimental conditions in Table 1.

### 2.3. Characterization

FT-IR spectra were recorded on a FT-IR spectrometer (8400S, Shimadzu, Kyoto, Japan) in the frequency range of 4000–500 cm^−1^ at a resolution of 4 cm^−1^. Subsequently, the structure of the samples was visualized by adopting a scanning electron microscope (Quattro S, Thermofisher, Waltham, MA, USA). The hydrogel samples were freeze-dried, brittle fractured with liquid nitrogen, and surface sprayed with gold. The thermal stability of the samples was measured by adopting a thermogravimetric analyzer (209F1, NETZSCH, Weimar, Germany). Around 10 mg samples were placed in an Al_2_O_3_ crucible and heated from room temperature to 600 °C at a rate of 10 °C/min under N_2_ atmosphere. By adopting an X-ray diffractometer (XRD-6100, Shimadzu, Japan) with a Ni-filtered Cu Kα (λ = 0.15418 nm) radiation, the XRD patterns of the samples were measured. The current is 30 mA, and the tube voltage is 40 kV. The diffraction angle (2θ) was scanned from 10° to 80° at 4°/min.

### 2.4. Swelling and pH Sensitivity

The swelling ratio of the hydrogels prepared in 0.9% NaCl solution and deionized water were determined gravimetrically. Approximately 100 mg of the hydrogels were subsequently immersed in an appropriate amount of 0.9% NaCl solution or deionized water at room temperature. Then, it was weighed immediately after removing the hydrogel from the swelling medium and wiping off the residual medium on its surface with a paper towel. By measuring the ESR of the hydrogels in solutions of different pH values, the pH sensitivity of the hydrogels was evaluated accordingly. The Formula (1) for calculating the equilibrium swelling ratio (*ESR*) is as follows:

(1) $$\;\;\;ESR=\frac{W_{e}-W_{0}}{W_{0}}$$

where *W*_0_ (g) and *W_e_* (g) are the mass before and after hydrogel swelling, respectively.

### 2.5. Adsorption Study

The adsorption experiments were performed by intermittent technique. Subsequently, deionized water was used to prepare a stock solution of MB at a concentration of 2000 mg/L, diluted appropriately to the desired concentration. The standard curve of MB was plotted in the concentration range 0–10 μg/mL. During the experimental tests, by adopting a UV-visible spectrophotometer (UV-2550, Shimadzu, Japan), the concentration of MB dye in all samples was measured at 664 nm [21], which was diluted to the range of 0–10 μg/mL. It is noteworthy that the 664 nm corresponds to the maximum absorbance of MB. The adsorption performance of C/G2 hydrogel on MB was tested under a distinct initial MB solute concentration (50–1250 mg/L), initial adsorbent dose (0.4–1.4 g/L), pH (3.0–9.0), temperature (30, 50, and 60 °C), and contact time (0–480 min) conditions. Furthermore, unless otherwise stated, experiments were maintained at an initial MB concentration of 500 mg/L, an adsorbent dose of 1 g/L, a pH of 7, and a temperature of 30 °C.

As a result of the following Formula (2), the equilibrium adsorption capacity *q_e_* (mg/g) can be calculated:

(2) $$q_{e}=\frac{\left(C_{0}-C_{e}\right)V}{W}\;\;\;\;\;$$

On the basis of the following Formula (3), removal (%) was calculated as follows:

(3) $$\;Removal=\frac{C_{0}-C_{e}}{C_{0}}\times 100$$

where *C_e_* (mg/L) is the equilibrium concentration and *C*_0_ (mg/L) is the initial concentration. *W* (g) is the weight of the adsorbent and *V* (L) is the volume of the solution.

### 2.6. Reusability of the Hydrogel

The MB-loaded hydrogel was stir-treated with 200 mL of 1 mol/L HCl for 5 h to desorb the MB dye. Subsequently, the hydrogels were washed with distilled water and then reused for adsorption processes again. The adsorption/desorption cycles were successively conducted three times, each trial with fresh solution.

## 3. Results and Discussion

### 3.1. Reaction Mechanism

**Figure 1:**
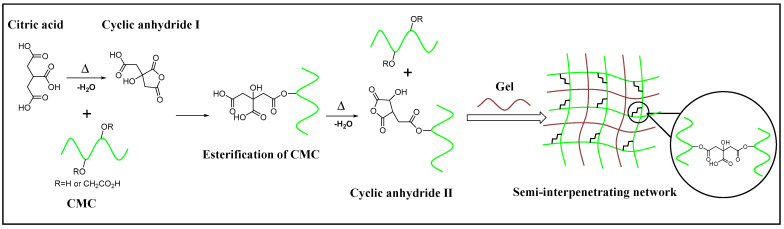
Schematic representation of the formation of hydrogel.

As conspicuously revealed in previous studies, citric acid cross-linking occurs in several stages [18,22]. The first step is that during the heating process, when a certain temperature is reached, citric acid undergoes intramolecular dehydration to form a more active five-membered ring anhydride. Subsequently, it speedily reacts with the hydroxyl group on the polysaccharide chain to form an ester bond. The remaining carboxyl groups then form another cyclic anhydride for further esterification and eventually form a diester bridge for cross-linking between polymer chains [23]. The hypothetical scenario for this mechanism is illustrated in Figure 1.

### 3.2. FT-IR Analysis

**Figure 2:**
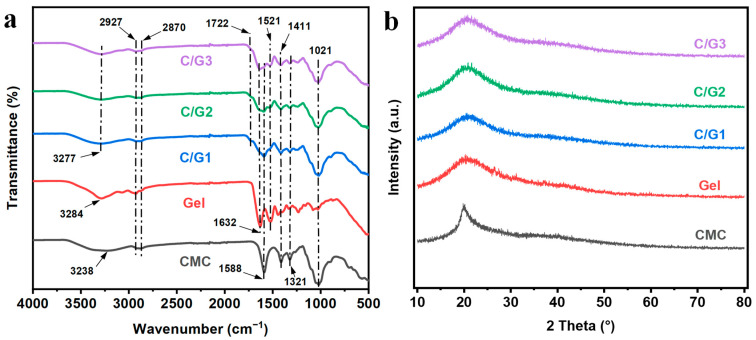
FT-IR spectra (**a**) and X-ray diffraction spectra (**b**) of the prepared hydrogels (C/G1, C/G2, and C/G3), and their initial components (CMC and Gel).

Figure 2a displays the FT-IR spectra of native Gel, CMC, and the prepared hydrogels (C/G1, C/G2, and C/G3). For CMC, the characteristic peaks at 3238, 2927, and 1588 cm^−1^ were attributed to O-H stretching vibration, CH_2_ asymmetric stretching vibration, and carboxylate C=O stretching vibration, respectively [24]. Other than that, the absorption peaks between 1200 and 1000 cm^−1^ stemmed from C-O-C stretching vibration [25]. The peaks at 3284 and 2870 cm^−1^ of the Gel can be corresponded to O-H stretching vibration, and C-H stretching vibration of the methylene groups [26]. The peaks at 1632 and 1521 cm^−1^ were due to the stretching vibrations of the C=O and -NH_2_ groups of the protein backbone [27]. As for the FT-IR spectra of C/G1, C/G2, and C/G3 hydrogels, the C=O stretching vibration peaks in CMC and -NH_2_ characteristic peaks in Gel are still present between 1021 and 1632 cm^−1^, but the peak shape changed substantially, and the peak intensity weakened. Thus, it can be assumed that there is some interaction between Gel and CMC, which makes the characteristic peak shape and intensity change [28]. Furthermore, a new peak was found at 1722 cm^−1^ after cross-linking, which corresponded with previously reported studies and was ascribed to the characteristic stretching band of the carbonyl group associated with esters bond formation, thus, the successful cross-linking of CMC molecular chains was confirmed in the hydrogel network [19]. Moreover, with the change of the Gel content in the composite hydrogel, the distance between CMC molecular chains increased and the degree of cross-linking decreased, resulting in weaker peak intensities.

### 3.3. XRD Analysis

XRD spectra for native Gel, CMC, and the prepared hydrogels (C/G1, C/G2, and C/G3) are exhibited in Figure 2b. CMC displayed a clear diffraction peak at 2θ = 20.0°, which arose from the semi-crystalline structure of CMC [29]. The diffraction peak of pure gelatin appeared at 2θ = 20.3°, which arose from the lateral stacking of the triple helix structure in the gelatin macromolecule [26], resulting in local order and displaying a certain degree of crystallinity. The diffraction peaks of C/G1, C/G2, and C/G3 hydrogels all appear around 2θ of 20.6°. Moreover, as demonstrated by our research results, these peaks become wider but less intense, which indicates a decrease in the overall crystallinity of the prepared semi-interpenetrating hydrogels. The reduction in crystallinity might be a result of the cross-linking reaction between citric acid and -OH in CMC, which triggered the formation of a mesh structure. Furthermore, there were the formation of hydrogen bonds between -OH in CMC and -NH_2_ in Gel. These not only limited the motility of CMC and gelatin molecules, but also destroyed the integrity of the molecular chains of CMC and gelatin, making the crystalline integrity of CMC and gelatin poorer and giving rise to the intensity (crystallinity) of the overall diffraction peak to decrease [30].

### 3.4. SEM Analysis

**Figure 3:**
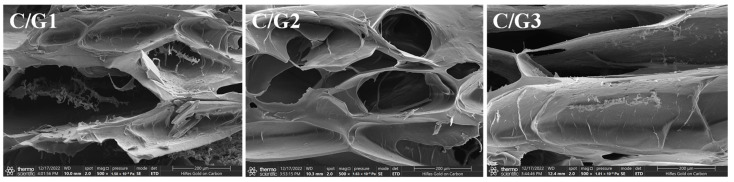
The SEM images of the C/G1, C/G2, and C/G3 hydrogels.

C/G1, C/G2, and C/G3 hydrogel SEM images are displayed in Figure 3. The surface of the C/G1 hydrogel was rough and irregular with incomplete pore structure. This is because the distance between the CMC molecular chains was very close, and the carboxyl groups on the CMC molecular chains would generate strong electrostatic repulsion between them, leading to the structural instability of the hydrogel and disintegration. With the increment of Gel content, the C/G2 hydrogel exhibited a relatively regular surface and a distinct porous network structure, evidencing uniform dispersion and strong interactions between the polymers. The structural stability of the hydrogel could be improved by adding an appropriate Gel to construct a semi-interpenetrating network. However, by further augmenting the Gel content, the surface of C/G3 hydrogel becomes rough and the pore structure becomes less and larger, which was not favorable for the adsorption of dyes. As a result, C/G2 hydrogel is comparatively more suitable for dye adsorption studies as a consequence of their relatively regular and distinct porous network structure, possessing a high surface area and providing large mass transfer channels [31].

### 3.5. Thermal Analysis

**Figure 4:**
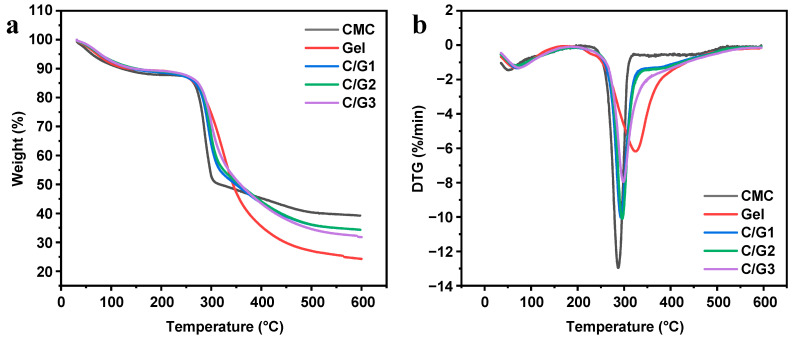
TGA (**a**) and DTG (**b**) curves of the prepared hydrogels (C/G1, C/G2, and C/G3), and their initial components (CMC and Gel).

As depicted in Figure 4, the thermal stability of pure CMC, pure Gel, the prepared hydrogels (C/G1, C/G2, and C/G3) were examined using thermogravimetric analysis (TGA/DTG). The thermogravimetric curves displayed two stages of CMC and Gel degradation and three regions of C/G1, C/G2, and C/G3 hydrogels degradation. The thermogravimetric analysis data of samples are displayed in Table 2. There is a first noticeable weight loss process in native CMC and native Gel at 35.5–120 °C and 35.5–165 °C, respectively, primarily attributed to the evaporation of physically adsorbed water molecules. In the second degradation region of CMC, mass loss was observed, which was the result of thermal degradation of its carboxyl groups (-COO^−^) structure [32]. Aside from that, the thermal decomposition of Gel gives rise to mass loss in the second stage. Thus, it is also worth noting that CMC is more thermally stable than Gel. The TGA/DTG curves of C/G1, C/G2, and C/G3 hydrogels revealed three main thermal degradation steps, which illustrates that multiple degradation processes coexisted. In comparison, the thermal stability of C/G1 and C/G2 hydrogels is basically the same, while the thermal stability of C/G3 hydrogel is somewhat lower [30]. The initial weight loss (11.72 wt% for C/G1, 11.28 wt% for C/G2, and 11.14 wt% for C/G3) was attributed to the evaporation of physically adsorbed water molecules. Moreover, the uppermost decomposition phases were found between 220 and 353 °C (C/G1 38.82 wt%, C/G2 38.24 wt%, C/G3 37.91 wt%), which could be ascribed to the breakage of the sugar ring backbone in CMC and the thermal decomposition of Gel [33].

### 3.6. Swelling Study

**Figure 5:**
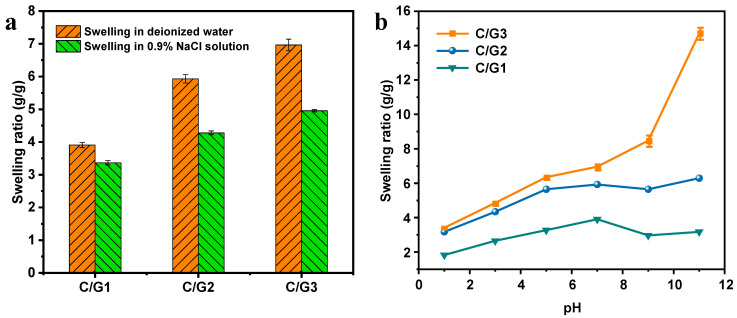
Swelling ratio of the prepared hydrogels (C/G1, C/G2, and C/G3) in distilled water and 0.9% NaCl solution (**a**) and the effect of pH on the swelling ratio of the prepared hydrogels (**b**).

The equilibrium swelling ratio of C/G1, C/G2, and C/G3 hydrogels in 0.9% NaCl solution and deionized water are displayed in Figure 5a. The equilibrium swelling ratio of C/G1, C/G2, and C/G3 hydrogels in deionized water, respectively, were 3.91, 5.93, and 6.97 g/g, which were slightly higher than those in 0.9% NaCl solution (3.37, 4.28, and 4.96 g/g, respectively). Moreover, the change of gelatin content could improve the swelling ratio of the prepared hydrogel; the predominant reason behind this may reside in that the increment of gelatin between CMC molecular chains hindered the cross-linking between citric acid and adjacent CMC molecular chains, lowering the extent of cross-linking and making the network structure of hydrogel looser, which increased the swelling ratio of hydrogel [34].

Figure 5b displays the results of the study on the effect of pH on the equilibrium swelling ratio of C/G1, C/G2, and C/G3 hydrogels. The swelling ratio of C/G1, C/G2, and C/G3 hydrogels were significantly higher in the pH range 1.0–7.0. This is due to the fact that under acidic conditions, the solution contained a large amount of H^+^ that could bind to -COO^−^, and the amino group in the form of -NH_3_^+^ that was electrostatically attracted to -COO^−^. These both inhibited the electrostatic repulsion between -COO^−^ groups, leading to the contraction of the network structure [35]. As the solution pH rises progressively, the electrostatic repulsion was promoted by the ionization of the -COOH groups, which advanced the swelling of the hydrogel network. In alkaline conditions, the swelling ratio of C/G1 and C/G2 hydrogels decreased, followed by an augment, which was triggered by the charge shielding effect of counter ions (Na^+^), so that the swelling ratio decreases [36]. Meanwhile, at pH = 11, the ester group formed between citric acid and CMC in the hydrogel would be hydrolyzed and broken in a strong alkaline environment so that the cross-linking degree decreased, and the network structure became loose, which would result in an increment in the swelling ratio. Moreover, the swelling ratio of C/G3 hydrogel has been exhibiting an increasing trend, which is due to the hydrolytic breakage of ester groups dominating the alkaline environment, lessening the extent of cross-linking and making the hydrogel of the network structure looser, giving rise to a noticeable augment in the swelling ratio. The results revealed that the prepared hydrogels were unsuitable for strong alkaline environments.

### 3.7. Adsorption of MB

**Figure 6:**
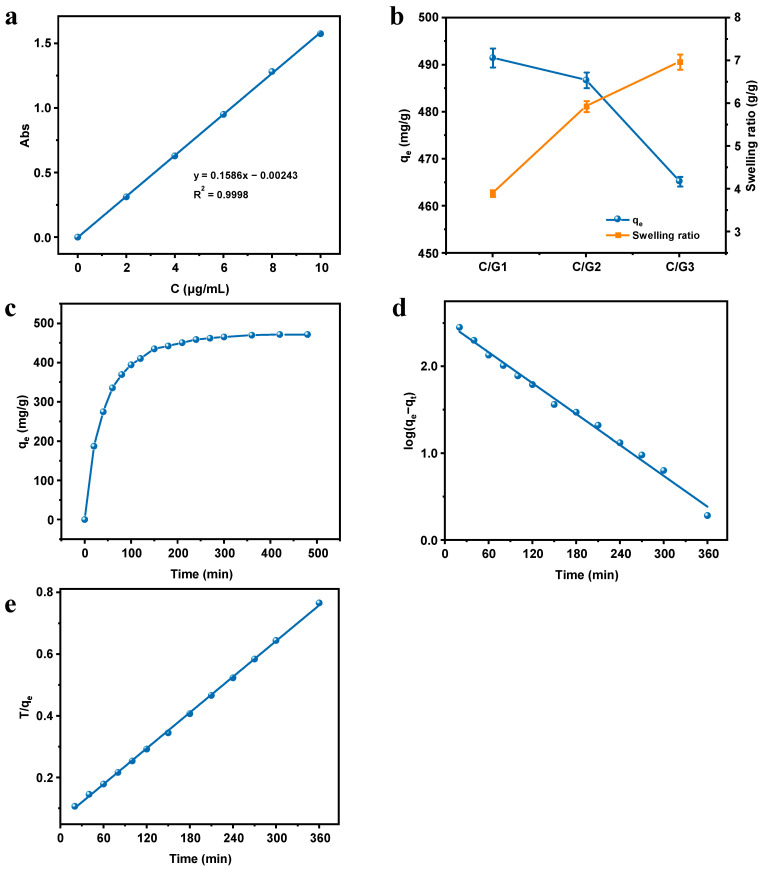
Standard curve of MB (**a**); The MB adsorption capacity of C/G1, C/G2, and C/G3 hydrogels at equilibrium and the swelling ratio in deionized water (**b**); the adsorption capacity of C/G2 hydrogel towards MB as function of contact time (**c**); Representations of the pseudo-first order (**d**) and pseudo-second order (**e**) kinetics models for the adsorption of MB by C/G2 hydrogel.

The absorbance A was plotted against the concentration C of MB solution. The standard curve was obtained by linear fitting. As shown in Figure 6a, the standard curve equation was y = 0.1586x − 0.00243 with R^2^ = 0.9998, and the correlation coefficient was high.

Figure 6b displayed the adsorption capacity of C/G1, C/G2, and C/G3 hydrogels at equilibrium and the swelling ratio in deionized water. As exhibited by the data analysis results, the adsorption capacity became smaller with the increment of the Gel content in the hydrogels, which might be a result from the reduction of CMC content per unit mass of hydrogel, resulting in the decrease of -COO^−^ as an active site. Meanwhile, in accordance with the previous discussion on hydrogel swelling, the higher swelling was favorable for the higher quality transport of MB within the hydrogels and shorter adsorption time [31]. Moreover, combined with the results of SEM and thermogravimetric analysis, we can draw a pertinent conclusion that the C/G2 hydrogel is more favorable for dye adsorption as a result of its superior pore size structure and better thermal stability. In summary, the C/G2 hydrogel was selected for the subsequent study of MB adsorption performance.

#### 3.7.1. Effect of Contact Time

In terms of adsorption, the contact time is crucial. In such a case, it is paramount to probe deep into the kinetics of adsorption and the equilibrium time. As a consequence, the effect of contact time on the adsorption MB by C/G2 hydrogel was tested systematically. The results of MB adsorption by the C/G2 hydrogel are described in Figure 6c. At the beginning, the adsorption ratio increased sharply, then gradually decreased, and reached an adsorption equilibrium after about 300 min. This might stem from the numerous free adsorption sites and the high MB concentration in the initial stage. At the equilibrium stage, the adsorption amount does not continue to increase because the adsorption and desorption of the pollutant ions by the adsorbent reach a dynamic equilibrium [37].

#### 3.7.2. Adsorption Kinetics

Using pseudo-first order (4) and pseudo-second order (5) kinetics models, the experimental data were fitted to identify the mechanism controlling adsorption:

(4) $$\log\left(q_{e}-q_{t}\right)=logq_{e}-\frac{k_{1}}{2.303}t$$

(5) $$\;\frac{t}{q_{t}}=\frac{1}{k_{2}q_{e}^{2}}+\frac{1}{q_{e}}t\;$$

where *q_t_* (mg/g) and *q_e_* (mg/g) represent the adsorption capacity of the adsorbent at time *t* (min) and at equilibrium, respectively. *k*_1_ (min^−1^) and *k*_2_ (mg/g/ min) are the rate constants of the pseudo-first and pseudo-second order kinetics models, respectively.

The obtained kinetics parameters and fit curves are displayed in Table 3 and Figure 6d,e, respectively. As evidently displayed by the research findings, the correlation coefficient (R^2^) obtained from the pseudo-first order model is lower than that obtained from the pseudo-second order model, which revealed that the pseudo-second order model matches the kinetics data better. Furthermore, the *q_e_* values received on the pseudo-first order model differed tremendously from the experimentally determined MB sorption values, but was fit with the *q_e_* values derived from the pseudo-second order model. To put it in another way, the pseudo-second order model dominated the MB adsorption by a C/G2 hydrogel, which suggests that the adsorption may be governed by a chemisorption process that predominantly involves the sharing or exchange of electrons between the hydrogel functional groups and the dye cations [38,39].

#### 3.7.3. Effect of the Initial MB Concentration

**Figure 7:**
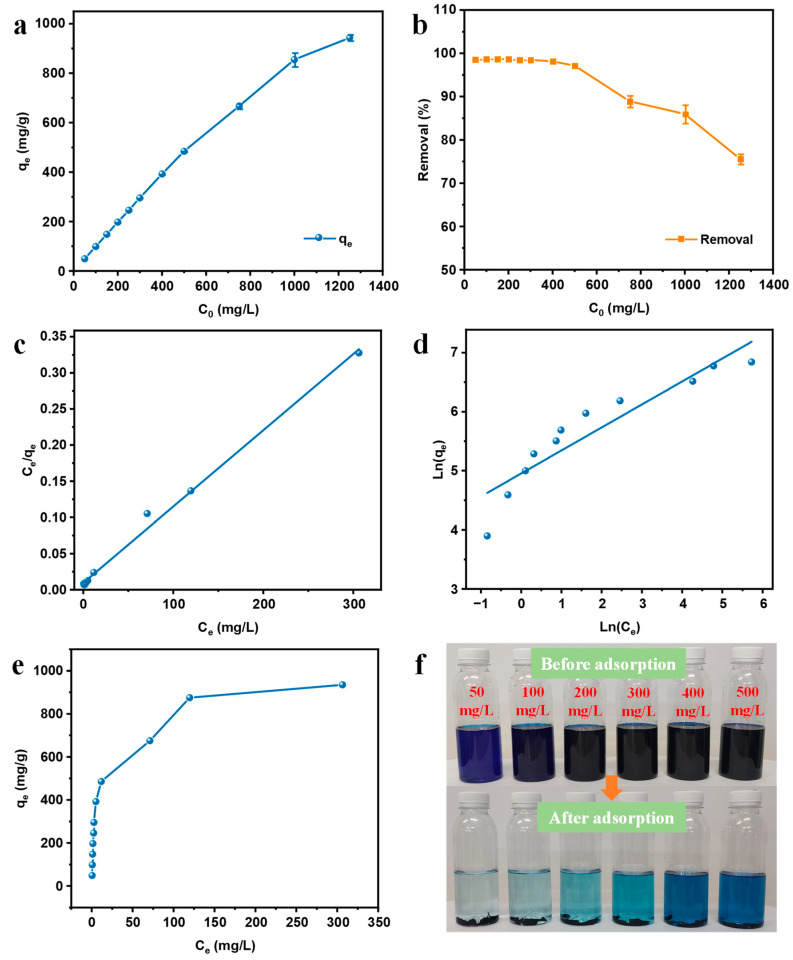
Adsorption capacities of C/G2 hydrogel at equilibrium versus initial MB concentration (**a**) and removal rate versus initial MB concentration (**b**); Langmuir (**c**), Freundlich (**d**), and adsorption isotherms (**e**) models for the adsorption of MB by C/G2 hydrogel; Images before and after adsorption of various concentrations of MB solution by C/G2 hydrogel (**f**).

At the equilibrium state, the adsorption of MB by C/G2 hydrogel at concentrations of 50–1250 mg/L was studied. As the MB concentrations rose continually, the adsorption capacity (q_e_) gradually increased (Figure 7a), but the removal ratio started to decrease remarkably when the initial MB concentration exceeded 500 mg/L (Figure 7b). The reason behind this may reside in that there are numerous adsorption sites available on the adsorbent at low concentrations. As the concentration of the initial MB solution heightens continuously, the adsorbent will occupy more adsorbed mass per unit mass as the competition for adsorption sites becomes more intense [40]. Nonetheless, the removal ratio will exhibit a decreasing trend.

#### 3.7.4. Adsorption Isotherms

As part of this study, Freundlich and Langmuir isotherm models were adopted to determine the C/G2 hydrogel of adsorption isotherm parameters. To be specific, the Freundlich isotherm model assumes that adsorption occurs on non-homogeneous surfaces and that the amount adsorbed heightens with the concentration through a multi-layer adsorption mechanism. Aside from that, the Langmuir isotherm model assumes the existence of a maximum limit absorption, which reflects the saturated mono-layer adsorption of adsorbate molecules on the adsorbent surface [41,42]. The linear form of Freundlich (6) and Langmuir (7) isotherm models can be expressed using the following equations:

(6) $$\;lnq_{e}=lnk_{F}+\frac{1}{n}lnC_{e}$$

(7) $$\;\;\;\frac{C_{e}}{q_{e}}=\frac{1}{k_{L}q_{m}}+\frac{1}{q_{m}}C_{e\;\;}$$

where *C_e_* is the concentration of MB at equilibrium in solution (mg/L), *q_m_* is the maximum adsorption capacity, *q_e_* is the equilibrium adsorption capacity of MB (mg/g), and *k_F_* and *k_L_* are the Freundlich constant and the Langmuir constant, respectively.

The Langmuir linear plots, Freundlich linear plots, and adsorption isotherm curve are depicted in Figure 7c–e. Table 4 presents the parameters of all isotherm models. Figure 7f exhibits the images of the solutions with diverse MB concentrations before and after adsorption by C/G2 hydrogel. It is noteworthy that the correlation coefficient (R^2^) obtained from the Freundlich model is noticeably lower than that of the Langmuir model, revealing that the Langmuir model is the best model to describe MB adsorption. Furthermore, the adsorption capacity of C/G2 hydrogel for MB from the experimental results was 941.25 mg/g, which nearly reached the theoretical maximum adsorption capacity (943.40 mg/g) from the Langmuir model. As evidently verified by the experimental results, the adsorption of MB was dominated by the Langmuir model, which demonstrates that the adsorption of MB by C/G2 hydrogel is saturated mono-layer adsorption.

Table 5 gathers the maximum adsorption capacity of different adsorbents cited in the literature. The maximum adsorption capacities of C/G2 hydrogel towards methylene blue is higher than those of all the listed adsorbents.

#### 3.7.5. Effect of Adsorbent Dosage

**Figure 8:**
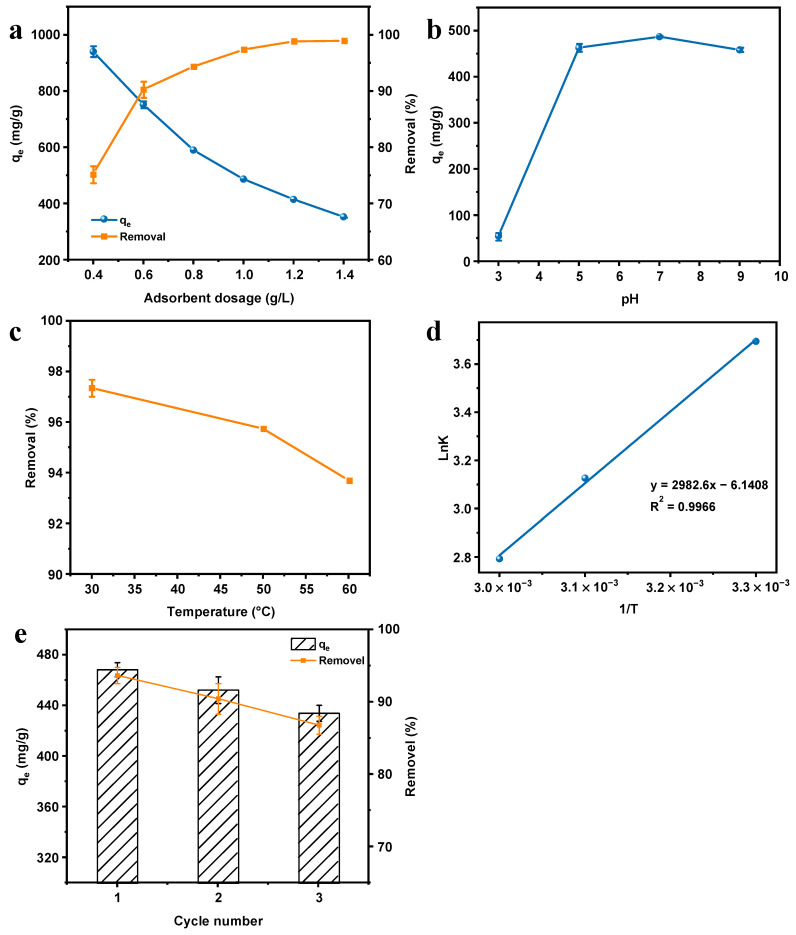
Effect of adsorbent quality on the removal efficiency and adsorption capacity of MB (**a**); Effect of pH on the adsorption of MB by C/G2 hydrogel (**b**); Effect of temperature on the removal efficiency (**c**) and thermodynamic fitting curve (**d**); Adsorption–desorption experiments of MB using C/G2 hydrogel (**e**).

Using adsorbent masses ranging from 0.4 g/L to 1.4 g/L, the effect of adsorbent dosage was evaluated. Figure 8a presents the results. With an augment in adsorbent mass from 0.4 g/L to 1.2 g/L, the removal percentage of MB increased from 75.24% to 98.85%. This behavior spawned from the fact that more adsorption sites are available to interact with dye molecules as the adsorbent dose rises continuously. Above this dose, the adsorption efficiency did not increase markedly, which might be a consequence of the saturation of the surface binding sites on the adsorbent. Moreover, with the increment of the dosage of the adsorbent, the adsorption capacity decreased strikingly. Similar findings have been reported in previous studies [19,45].

#### 3.7.6. Effect of pH

As is known, an essential factor that affects the adsorption process is the solution pH [46]. The change of pH will not only affect the physicochemical properties of the adsorbate molecules or ions and the adsorbent, but also affect the degree of ionization of functional groups on the adsorbent surface [47]. As a result, as displayed in Figure 8b, the adsorption of MB by C/G2 hydrogel was investigated under various pH conditions. As demonstrated by the experimental results, the adsorption exhibited a trend of increasing and then decreasing as the pH heightens. Since adsorption is an electrostatic attraction between the anionic active sites on the adsorbent surface and MB cations, the acidic environment inhibits the ionization of -COOH groups, and the adsorption capacity gradually increases as the degree of ionization rises with the increment of pH to produce more active sites. It deserves to be mentioned that the adsorption capacity reached the maximum in neutral solution. In an alkaline environment, the active sites were partially lessened owing to the shielding effect of counter ions (Na^+^) and the adsorption capacity displayed a downward trend [43].

#### 3.7.7. Effect of Temperature

The adsorption capacity is also affected by the temperature of the solution. The adsorption of MB by C/G2 hydrogel at three temperatures (30, 50, and 60 °C) was investigated under the same other conditions. The results are displayed in Figure 8c. The removal ratio decreased from 97.35% to 93.70% as the temperature rises continuously, which indicated that it was an exothermic process when MB was adsorbed by C/G2 hydrogel [25].

To effectively evaluate the effect of temperature variation on the adsorption of MB by C/G2 hydrogel and better understand this process, by adopting the van’t Hoff Equation (8), various thermodynamic parameters (Δ*H*, Δ*S*, Δ*G*) were estimated as follows:

(8) $$\;lnK=-\frac{\Delta G^{{}^{\circ}}}{\mathrm{RT}}=-\frac{\Delta H^{{}^{\circ}}}{\mathrm{RT}}+\frac{\Delta S^{{}^{\circ}}}{R}$$

where R is the general gas constant (8.314 × 10^−3^ kJ·mol^−1^·K^−1^), *K* =$\;\;\frac{Q_{e}}{C_{e}}$ is the equilibrium constant or the linear adsorption distribution coefficient, and T is the temperature of solution (K). Δ*G°*, Δ*S°*, and Δ*H°* are the standard Gibbs free energy change, the standard entropy change, and the standard enthalpy change.

On the basis of the slope and intercept of the fitted curve (Figure 8d), Δ*S* and Δ*H* can be estimated, and Δ*G* is further obtained. The values are displayed in Table 6. At all temperatures studied, Δ*G* was negative, proving a favorable and spontaneous adsorption of MB by the C/G2 hydrogel. With the augment in temperature, the value of Δ*G* also increased, which brought about a negative value of Δ*H*, revealing that the adsorption of MB by C/G2 hydrogel was exothermic. It corresponds with the fact that as the temperature rises progressively, the removal ratio decreases [44].

#### 3.7.8. Reusability of the Prepared Hydrogel

Figure 8e shows the experimental results of the recycling of the C/G2 hydrogel for MB adsorption. After three adsorption–desorption processes, the equilibrium adsorption capacity of the hydrogel decreased from 468.25 mg/L to 434.00 mg/L, and the removal rate decreased from 93.65% to 85.92%, which was because the adsorption of MB by C/G2 hydrogel was chemisorption, and when some of the adsorption sites on the adsorbent were occupied by MB molecules, the adsorbent and adsorbent mass combination was more stable, resulting in some MB being difficult to elute down, so the removal rate decreased. After three adsorption–desorption processes, the removal rate of MB by C/G2 hydrogel still reached more than 85%, indicating that the hydrogel could still effectively remove MB from an aqueous solution after repeated use.

## 4. Conclusions

By adopting the solution casting method in which citric acid was treated as a cross-linking agent, environmentally friendly sodium carboxymethyl cellulose/gelatin semi-interpenetrating hydrogels were synthesized in this study. XRD, FT-IR, SEM, and TG/DTG were used to characterize and analyze the raw materials and the prepared hydrogels. The results showed that the citric acid successfully cross-linked the CMC molecular chains; the C/G2 hydrogel had a relatively regular and obvious porous network structure with good thermal stability and was suitable as a material for dye adsorption. The swelling performance study showed that the prepared hydrogels were pH sensitive and salt ion sensitive. The adsorption study of C/G2 hydrogel for MB showed better adsorption conditions: the adsorption time was 300 min, the adsorbent dosage was 1.2 g/L, the initial concentration of MB was 500 mg/L, the adsorption temperature was 30 °C, and the pH value was 7. The adsorption kinetics study was in accordance with the pseudo-second order kinetics model, indicating that the adsorption process was controlled by chemisorption. The adsorption isotherm study was in accordance with the Langmuir model, indicating that the adsorption process was monolayer adsorption with a maximum equilibrium adsorption capacity of 943.15 mg/g. The thermodynamic study showed that the adsorption process was spontaneous, exothermic, and entropy decreasing. Repeatability experimental studies showed that after three adsorption–desorption cycles, the removal of MB by C/G2 hydrogel still reached 85% with good reusability performance. As a consequence, the prepared hydrogels, especially the C/G2 hydrogel, can be employed as environmentally friendly, efficient, and stable adsorbents for dye wastewater treatment.

## Figures and Tables

**Table 1:** Preparation conditions of C/G1, C/G2, and C/G3 hydrogels.

Hydrogel Code	CMC (g)	Gel (g)	Citric Acid (g)
C/G1	3	1	0.135
C/G2	3	2	0.135
C/G3	3	3	0.135

**Table 2:** TGA and DTG data for CMC, Gel, C/G1, C/G2, and C/G3 hydrogels.

Sample	Decomposition Stage	T_max_ (°C)	Weight Loss (%) Partial	Total
CMC	1st	51	12.24	60.74
	2nd	287	48.50	
Gel	1st	69	11.12	75.31
	2nd	324	64.19	
C/G1	1st	70	11.72	65.68
	2nd	293	38.82	
	3rd	371	15.14	
C/G2	1st	70	11.28	65.63
	2nd	296	38.24	
	3rd	374	16.11	
C/G3	1st	71	11.14	67.84
	2nd	298	37.91	
	3rd	378	18.79	

T_max_: Temperature of maximum weight loss.

**Table 3:** Parameters of different kinetics models for MB adsorption by C/G2 hydrogel.

Pseudo-First Order			Pseudo-Second Order		
*k*_1_ (min^−1^)	*q_e_* (mg/g)	R^2^	*k*_2_ (g/mg/min)	*q_e_* (mg/g)	R^2^
0.0137	329.84	0.9926	5.89 × 10^−5^	518.13	0.9995

**Table 4:** Parameters of Langmuir and Freundlich isotherm models for MB adsorption by C/G2 hydrogel.

Langmuir Model			Freundlich Model		
q_max_ (mg/g)	K_L_ (L/mg)	R^2^	k_F_ (mg/g)	n	R^2^
943.40	0.1118	0.9942	141.95	2.5660	0.8508

**Table 5:** Comparison of the maximum adsorption capacity of the present study with that previously reported.

Adsorbent	q_e_ (mg/g)	Reference
CMC	25	[18]
CMC-HEC	769.23	[19]
CS/CMC-PEG	331.72	[39]
Hyd/CB	27.32	[40]
CMC-PUF-10	27.50	[41]
CMC-Alg/GO	78.50	[42]
CMC/CS	120	[43]
CMC-g-poly(AA-co-IA)/MMT	19.12	[44]
C/G2	943.40	This work

**Table 6:** Thermodynamic parameters for the adsorption of MB by C/G2 hydrogel.

T (K)	∆*G* (kJ·mol^−1^)	∆*H* (kJ·mol^−1^)	∆*S* (J·mol^−1^·K^−1^)
303	−9.31		
323	−8.40	−24.80	−51.05
333	−7.73		

## Data Availability

Data sharing is not applicable to this article.
